# Impacts of Land Use Changes on Soil Properties and Processes

**DOI:** 10.1155/2014/831975

**Published:** 2014-12-31

**Authors:** Antonio Paz González, Cleide Aparecida de Abreu, Ana Maria Tarquis, Eduardo Medina-Roldán

**Affiliations:** ^1^Soil Science Department, Faculty of Sciences, University of A Coruña (UDC), 15008 A Coruña, Spain; ^2^Center for Soils and Environmental Resources, Agronomic Institute of Campinas (IAC), Avenida Barão de Itapura 1481, 13022902 Campinas, SP, Brazil; ^3^Applied Mathematics Department, Polytechnical University of Madrid, Ciudad Universitaria, s/n, 28040 Madrid, Spain; ^4^CEIGRAM, Polytechnical University of Madrid, Ciudad Universitaria, s/n, 28040 Madrid, Spain; ^5^Department of Environmental Sciences, Xi'an Jiaotong-Liverpool University, Suzhou, Jiangsu 25123, China

Land use is one of the main drivers of many processes of environmental change, as it influences basic resources within the landscape, including the soil resources. Poor soil management can rapidly deteriorate vast amounts of land, which frequently becomes a major threat to rural subsistence in many developing and developed countries. Conversely, impact of land use changes on soil can occur so unnoticed that land managers hardly contemplate initiating ameliorative measures. Knowledge and understanding of soil properties and processes ensures remediation or reclamation of disturbed or damaged soils.

This special issue brought together an international group of scientists presenting results from field trials and data harvesting carried out in a range of different soils and environments, from Poland, Italy, Spain, and USA to China, Indonesia, Venezuela, Brazil, and Argentina, together with laboratory experiments, reviews, and modeling with advanced mathematical tools. The strength of such issue was derived from a mutual interest in the mechanisms that regulate the impact of land use and changes in land use on soil properties and processes and also in the development and use of the most advanced methods and procedures for assessing them.

Drawing on the latest research and opinion, first this issue contains one state-of-the-art review and two research articles highlighting the usefulness and efficiency of the approach adopted here in a general context. W. Zhou et al. reviewed the effect of paddy upland rotation on soil properties. W. Shangguan et al. addressed the soil pedodiversity of China, mapping the distribution and extent of different soil taxa; this allowed identification of nearly 90 endangered soils, also suggesting that at least two dozens of soils have already gone extinct due to inadequate land use. J. Rejman et al. addressed the role of land use change in soil losses and relief modification in Loess areas of Poland.

Various authors reported laboratory experiments aiming to clarify the role of external inputs (amendments, irrigation, etc.) in selected soil properties. More specifically, A. D. Karathanasis et al. used several amendment materials together with extracts from crop biomass to accelerate fragmentation of fragipans and, therefore, to increase the water holding capacity of these soils. L. Chu et al. assessed the potential of microsprinkler irrigation as a method to alleviate soil salinization, allowing crop growth. M. García-Albacete et al. conducted leaching experiments to analyse phosphorus mobility in both soil-compost ad soil-digestate systems, showing that phosphorus losses were higher for the former than for the latter; in addition this study provided evidence of the importance of waste's wettability for assessing P sorption mechanisms and risk of leaching losses. M. García-Sánchez et al. using a batch experiment showed that both organic amendments and a sulfur compound added to two different Hg contaminated soils (luvisol and chernozem) were able to reduce Hg mobile fractions and increased availability of macro- and micronutrient.

The role of land use in soil organic matter and nitrogen dynamics has been illustrated by case studies carried out in contrasting soil and climatic conditions. A. F. González-Pedraza and N. Dezzeo studied soil nitrogen seasonality in the Western Llanos of Venezuela. S. Dori et al. addressed soil carbon dynamics in different regions of Europe and discussed the efficiency of management practices that control the potential of sequestration of soil organic carbon. A. Liang et al. analysed the mechanisms of soil organic carbon turnover on agricultural systems using a combination of isotopic tracer and physical fractionation under no-tillage and mouldboard ploughing; results showed that shot term impact of the studied tillage treatments varied in the different fractions analysed. D. A. McGranahan et al. analysed the reliability of carbon sequestration estimations associated with the effect of unexplained variability and due to interactions of vegetation, land use management, and soil properties with belowground ecosystem function; subsequently, even if rangeland soils are important carbon pools, it is unlikely that rangeland plant communities can be effectively categorized by their carbon sequestration potential.

Land use impacts on greenhouse gasses have been a major topic of this special issue. Z. S. Zhang et al. conducted a field trial to evaluate the effect of mulching from residues of a previous crop on paddy fields under no-tillage; this management system was found to significantly increase CO_2_ and N_2_O emissions, while decreasing CH_4_ emissions. S. F. Smith and K. R. Brye reported results from a field trial on a silt loan soil under soybean, showing that the impact of irrigation on seasonal CO_2_ emissions differed between years, whereas no-tillage management reduced seasonal CO_2_ emissions; the tillage effect on total CO_2_ emissions was not dependent on the irrigation scheme used. L. N. L. K. Choo and O. H. Ahmed used a lysimeter experiment to analyse both CO_2_ emissions and dissolved organic carbon leaching in a drained peat land cropped to pineapple under tropical conditions. Y. Lu and H. Xu performed an incubation experiment to test the effects of soil temperature, flooding, and organic matter addition on N_2_O emissions in a wetland soil.

Two manuscripts addressed the role of land use in soil organism. G. M. Siqueira et al. used the classical pitfall trap method to study the interactions between the soil arthropod community and land use and management of an entisol under semiarid climate in Brazil; the arthropod abundance under native forest was much lower than under native biomes with tropical climate. Agricultural land use strongly decreases the abundance of Formicidae compared to natural biome. E. E. Kuramae et al. studied the role of several land uses in the structure and composition of microbial communities in Netherlands using DNA analysis; the functional gene diversity found in different soils did not group the sites accordingly to land management, and the main factors driving differences in functional genes between land uses or management systems were carbon : nitrogen ratio, phosphatase activity, and total nitrogen.

New experimental and conceptual methods are needed to assess the effects of land use changes on soil properties and processes. J. L. M. P. de Lima et al. mapped soil surface macropores using infrared thermography; this technique is expected to provide a better understanding of the complex relationships between soil pores and soil physical and hydraulic properties. C. Moreno et al. developed an image analysis method to estimate the soil cover by different types of mulching materials during degradation in the field; particular attention was paid to thresholding methods in image treatment; proportion of areas lacking mulch have been automatically assessed. The applications of fractals and multifractals in soil and earth sciences are increasing, since many soil properties and processes have been shown to depend on complex interactions that could be assessed by fractal models. Also there is an increasing availability of data sets allowing computation and modelling using these mathematical tools. J. de Castro et al. described fractal analysis of Laplacian pyramidal and applied this method to segmentation of soil micromorphology; the algorithm used produced more reliable results than the commonly employed OTSU algorithm.

Geostatistics was used to evaluate the spatial variability of several soil properties as related to land use at various sampling scales. G. M. Siqueira et al. used soil apparent electrical conductivity for devising soil sampling schemes in an agricultural field that in a further step were analyzed by geostatistical techniques; as a result, a first manuscript was devoted to estimated spatial patterns of soil compaction and a second one provided insight into the spatial variability of selected general soil properties. L. A. Morales et al. studied the spatial distribution of ammonium-nitrogen, phosphorus, and potassium in a paddy field at Argentina during three different vegetative periods of the rice crop. X. Tan et al. analyzed the spatial variability of sixteen soil properties, including several soil enzymes, focusing on soil quality assessment; it was concluded that the spatial patterns of soil quality were better reflected using an integrated index based on soil enzyme activities. P. L. Aguado et al. used multifractal analysis to characterize a landscape, based on a high resolution digital elevation model; it was shown that the use of the multifractal approach with mean absolute gradient data is a useful tool for analyzing topographical features.

We believe that the present special issue reflects recent advances on the effects of land use over a range of soil properties and processes, complemented with insightful case studies using advanced mathematical techniques and new experimental methods for assessing soil surface characteristics.

